# Modified suture-assisted canaloplasty in Asians with primary open-angle glaucoma: a prospective study with 12-month follow-up

**DOI:** 10.1186/s12886-022-02424-9

**Published:** 2022-05-04

**Authors:** Ya Liang, Qiuli Yu, Hong Sun, Sucijanti, Liuwei Gu, Zhilan Yuan

**Affiliations:** 1 Department of Ophthalmology, the First Affiliated Hospital of Nanjing Medical University, No. 300 Guangzhou Road, Nanjing, 210029 China; 2 Department of Ophthalmology, the Second Affiliated Hospital of Nanjing Medical University, No.262 North Zhongshan Road, Nanjing, 210003 China

**Keywords:** Canaloplasty, Suture, Viscocanalostomy, Schlemm’s canal, Primary open-angle glaucoma

## Abstract

**Background:**

To evaluate the efficacy and safety of modified suture-assisted canaloplasty in Asians with primary open-angle glaucoma (POAG).

**Methods:**

A prospective, consecutive cases study, evaluating a modified canaloplasty by twisted 6/0 suture was performed on Asian POAG patients. Three modifications of this canaloplasty included opening the Schlemm’s canal by viscocanalostomy, circumferential probing by a twisted 6/0 suture and loose suture of the superficial scleral flap. The twisted 6/0 suture was selected as a prober based on characteristical analysis of size and contact measurement as well as chemical composition conducted among 5/0, twisted 6/0 polypropylene sutures and the microcatheter. Success criteria were defined as intraocular pressure (IOP) ≤ 21 mmHg, 18 mmHg, 15 mmHg, and ≥ 20% reduction without (complete success) or with medications (qualified success). Efficacy was assessed by the success rate of circumferential catheterization, IOP values, the success rate of the surgery, the number of IOP-lowering medications, best-corrected vision acuity (BCVA), cup-to-disc ratio (C/D), and mean deviation (MD). Safety was evaluated by adverse events.

**Results:**

Forty eyes from 40 consecutive patients were included with a mean follow-up of 14.8 ± 3.0 months. Circumferential catheterization was successfully conducted in 36 eyes (90%). Mean IOP decreased from 26.2 ± 6.9 mmHg to 14.5 ± 2.7 mmHg at 12 months postoperatively. While medication numbers were reduced from 3.2 ± 0.6 to 0.5 ± 0.8 at month 12 (both *p* < 0.001). Qualified success rate was 97.2% [95% confidence interval (CI) 0.92–1.03], 86.1% (95% CI 0.74–0.98) and 66.7% (95% CI 0.51–0.83) at 12 months with three criteria. BCVA, C/D and MD did not show progression at 1-year follow-up (*p* > 0.05). Age, baseline IOP, and spherical equivalent negatively influenced the success rate significantly (all *p* < 0.05). Adverse events included hyphema (30.6%), IOP spike > 25 mmHg (8.3%), and peripheral synechia to the trabecular-Descemet’s membrane (2.7%).

**Conclusion:**

Twisted 6/0 suture can be an ideal material for cannulation. Modified suture-assisted canaloplasty is an effective, safe alternative with a cost-efficient feature for patients with POAG, especially in developing countries.

**Trial registration:**

This trial was registered in the Chinese Clinical Trial Registry (ChiCTR1900028618, 29/12/2019).

**Supplementary Information:**

The online version contains supplementary material available at 10.1186/s12886-022-02424-9.

## Background

The pathogenesis of primary open-angle glaucoma (POAG) is the resistance in the juxtacanalicular meshwork and inner wall of Schlemm’s canal (SC) and canal collapse [1]. Thus, viscocanalostomy is applied in POAG patients to restore the physiological outflow drainage by relieving the resistance and dilating SC with a 120-degree range [2]. Compared with trabeculectomy, viscocanalostomy has a good safety profile and a less intraocular pressure (IOP)-lowering effect [3]. The range of SC dilation correlates with IOP reduction. In 2007, Lewis et al. accomplished canaloplasty for the first time by an illuminated microcatheter (iTrack™, Ellex, Menlo Park, California, USA) [4]. Canaloplasty incorporates circumferential dilation by viscoelastic agent and suture distension of SC. Several studies show that canaloplasty lowers IOP by 2 mmHg than viscocanalostomy [5, 6].

However, the illumination equipment is so expensive that most patients cannot afford undergoing surgery, especially in developing countries. Furthermore, we previously reported that the suture was superior to the microcatheter in terms of flexibility for probing of SC[7]. Smith firstly tried a fine nylon suture to traverse almost half the circumference of the SC[8]. Beck first applied a 6/0 polypropylene suture in circumferential cannulation in trabeculotomy for primary congenital glaucoma (PCG) in 1995 [9]. In 2019, Haus and Kodomskoi proposed polypropylene sutures instead of the microcatheter for circumferential probing in canaloplasty [10, 11].

Although suture-assisted canaloplasty was reported in Whites and Blacks with POAG [12, 13], the efficacy and safety were still unknown for Asian patients. Based on our experience of viscocanalostomy over two decades [14], SC could be accurately opened and located, which is a prerequisite for circumferential cannulation. Our previous study applied suture-assisted trabeculotomy in PCG, and the success rate of circumferential catheterization was 84.8% [15]. The evidence remains unknown whether suture-assisted canaloplasty is indicated for adult patients with POAG. Thus, we developed a new, modified, and cost-efficient procedure. First, viscocanalostomy was performed, and SC was opened accurately during dissection of the deep scleral flap. Second, a twisted 6/0 polypropylene suture was used for circumferential probing of SC. Third, the superficial scleral flap was loosely sutured for external outflow of aqueous humor.

Here, we present 12-month efficacy and safety results of modified suture-assisted canaloplasty in a prospective consecutive series of Asian POAG patients. Moreover, we conducted characterization comparison among 5/0, twisted 6/0 sutures, and the microcatheter.

## Materials and methods

### Characteristical comparisons of cannulation materials

**Fig. 1:**
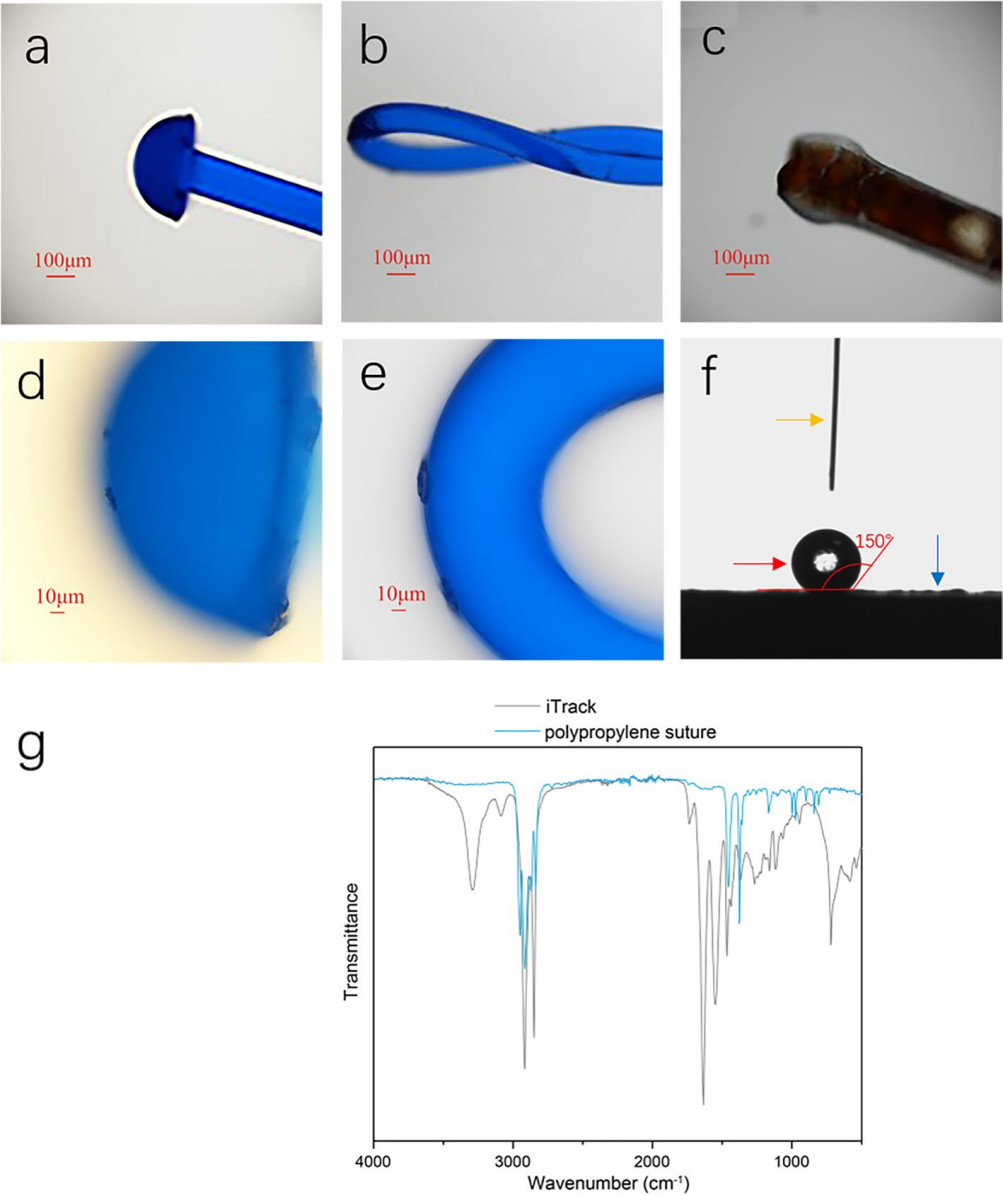
Characterization of cannulation materials. **a** 5/0 polypropylene suture with a mushroom-shaped tip. **b** Twisted 6/0 polypropylene suture with a loop tip. **c** iTrack™ with an inflated tip. **d** 5/0 polypropylene suture with a rough and sharp margin. **e** Twisted 6/0 polypropylene suture with a smooth tip. **f** The measurement of contact angle. A 5 µl droplet (red arrow) of distilled water from a very thin tube (yellow arrow) was used for contact angle measurement of polypropylene sutures (blue arrow). The angle (red angle) between the droplet (red arrow) and the surface of the suture (blue arrow) was 150°, which indicated the polypropylene was hydrophobic and stable in water. **g** Fourier transform infrared (FTIR) spectra of the polypropylene suture and iTrack™

A twisted 6/0 polypropylene suture (Prolene®, Johnson and Johnson Medical GmbH Ethicon, Norderstedt, Germany) was selected for the cannulation of SC in the modified technique based on the analysis of several candidates, including 5/0 polypropylene sutures, twisted 6/0 polypropylene sutures, and iTrack™. The diameter and morphology of three candidate fibers were photographed using an optical microscope (Olympus-BX53, Japan). The illuminating source was white light (Fig. 1a-e). Contact angle measurements were carried out on a contact angle goniometer (KSV CAM 101, Finland). A 5 µl droplet of distilled water was used for the measurement. Sliding angles between the droplet and the surface of the suture were measured using a purpose-made device consisting of a sample holder and a digital angle meter (Fig. 1f). Fourier transform infrared (FTIR) spectra were obtained on a Bruker (Alpha) FTIR spectrometer to analyze the chemical composition of polypropylene sutures and iTrack™ (Fig. 1g).

### Patients

A prospective cohort study was conducted on 40 patients attending a single ophthalmological department from December 30, 2019, to July 2, 2020. All patients were followed up for at least 12 months. The study followed the tenants of the Declaration of Helsinki and was approved by the Ethics Committee of the First Affiliated Hospital of Nanjing Medical University (No.2019-SR-457). This trial was registered in the Chinese Clinical Trial Registry (ChiCTR1900028618, 29/12/2019). Written consent was obtained from all patients before surgery.

Patients needed to meet all inclusion criteria which were as follows: (1) POAG with IOP > 21 mmHg on maximal anti-glaucoma medications; (2) glaucomatous optic disc neuropathy and visual field deficiency; (3) wide anterior chamber angle with distinct trabecular meshwork by gonioscopy (Ocular®, Single mirror gonio diagnostic lens, Bellevue, U.S.A.). Exclusion criteria included: (1) secondary glaucoma; (2) combined with phacoemulsification; (3) history of glaucoma surgery. After admission, baseline data were collected as follows: IOP values, best-corrected visual acuity (BCVA), refractive errors, cup-to-disc ratio (C/D), median deviation (MD), number of anti-glaucoma medications, and complications.

### Surgical technique

**Fig. 2:**
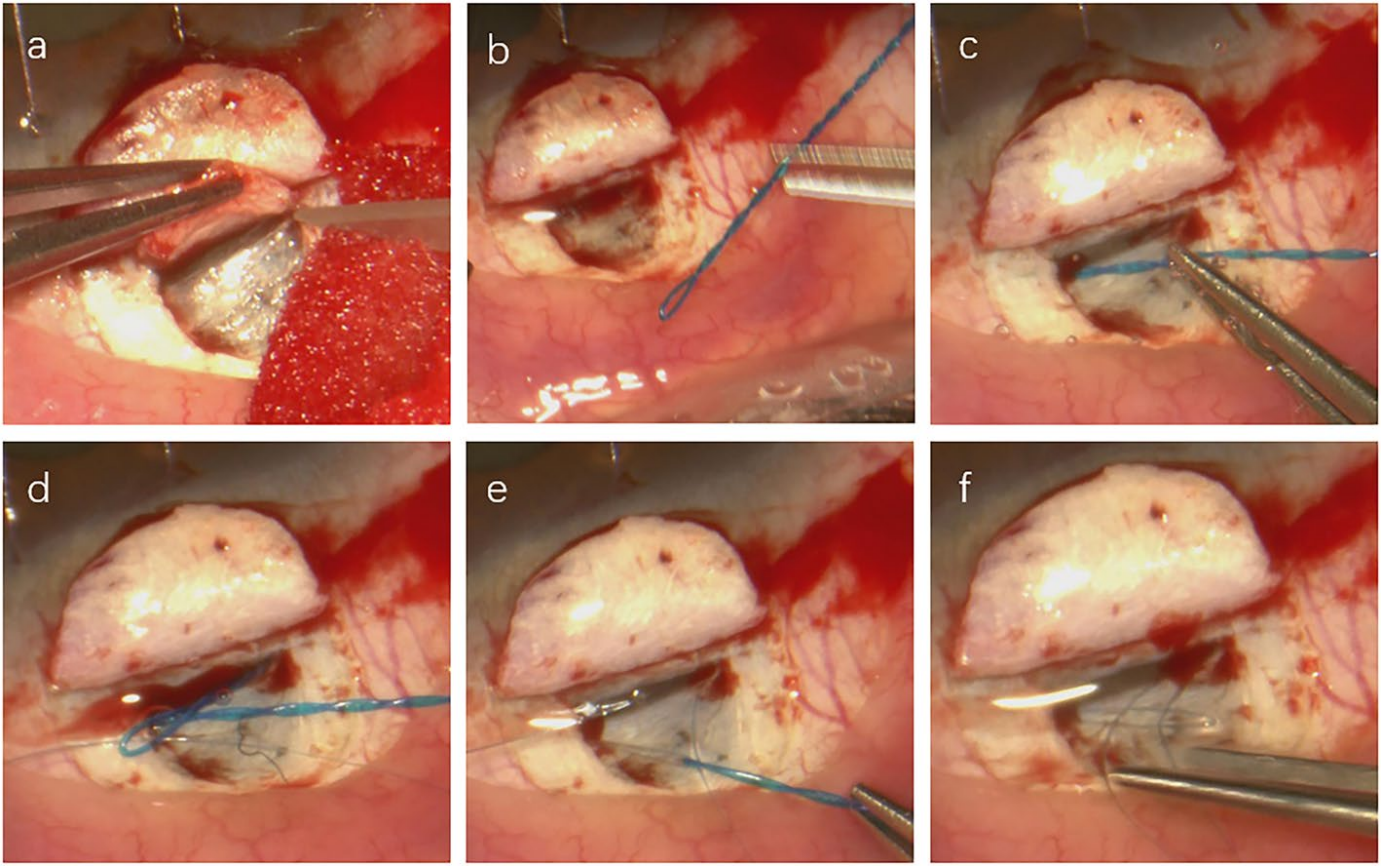
Surgical procedure. **a** A superficial and parabolic scleral flap was made, and a deep scleral flap of 3 × 3 mm was dissected, opening Schlemm’s canal(SC). **b** A twisted 6/0 polypropylene suture was prepared. **c** The loop tip was inserted into one ostium of SC. **d** The tip came out from the other ostium through 360-degree advancement. The loop tip was then wrapped with the 10/0 suture. **e** The 6/0 suture was withdrawn and replaced by a twin 10/0 suture in SC. **f** The 10/0 suture was tied with a tension distending the trabecular meshwork

Solo modified canaloplasty was performed by a senior doctor. Figure 2 exhibits the whole process. First, the conjunctiva and Tenon’s capsule were opened. A superficial parabolic scleral flap of 5 × 5 mm and one-third thickness was dissected and lifted towards the limbus. A deep flap of 3 × 3 mm was created with glimpsed choroid under the sclera until the scleral spur. Next, the scleral spur was carefully separated, and SC was accurately opened just behind the scleral spur. The trabecular-Descemet’s membrane (TDM) was exposed, and both ostia were created. A viscoelastic (Hyaluronic acid Healon GV®, Abbott Medical Optics Inc., USA) was injected into both ostia five times respectively by a specially made needle [14]. SC was dilated, facilitating suture probing afterward. The viscoelastic may be pushed forward by the tip of the suture; thus, the whole SC and distal outflow way of the aqueous humor could be dilated during suture cannulation [7]. Then, a twisted 6/0 polypropylene suture was made. Atraumatic probing of SC was ensured because of the smooth and loop tip. The loop tip was inserted from one ostium and came out from the other ostium through 360-degree probing. The surgeon could feel resistance during suture cannulation. Bleeding was usually observed in the exit ostium before the suture came out. A 10/0 polypropylene suture (Prolene®, Johnson and Johnson Medical GmbH Ethicon, Norderstedt, Germany) was threaded through the loop tip of the 6/0 suture, which was withdrawn and replaced by a twin 10/0 suture in SC. The 10/0 suture was tied using three knots with inward distension. In the end, both the superficial scleral flap and conjunctival flap were sutured with a 10/0 nylon suture (Ethilon®, Johnson and Johnson Medical Ltd, Livingston, UK). The superficial scleral flap was sutured loosely with two sutures. Healon GV® was injected under the superficial scleral flap to maintain the scleral lake.

All patients received miotic drops (0.5% pilocarpine) for two months postoperatively.

**Fig. 3:**
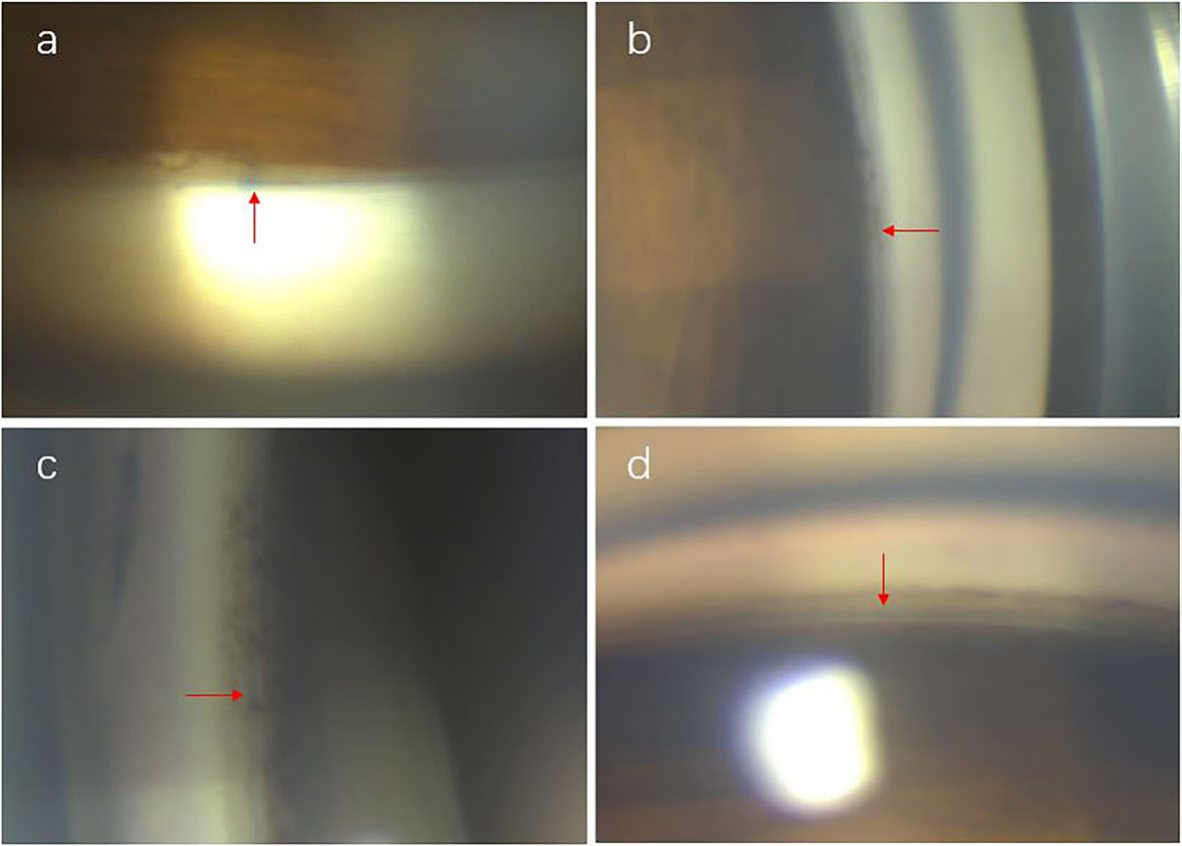
Postoperative view of gonioscopy. Red arrows indicate the 10/0 suture. **a** Superior quadrant. **b** Nasal quadrant. **c** Inferior quadrant. **d** Temporal quadrant

A compound medicine of tobramycin and dexamethasone (0.3% tobramycin, 0.1% dexamethasone) was prescribed for only one week to avoid steroid-induced ocular hypertension, and then replaced by topical antibiotics (0.3% ofloxacin) for two weeks and nonsteroidal anti-inflammatory eye drops (0.1% pranoprofen) for two months. Gonioscopy was used to make sure that the suture was in SC. The view from the gonioscope is shown in Fig. 3.

### Follow-up

The following baseline parameters were recorded: IOP measurement, number of anti-glaucoma medications, and complications following postoperative visit: day 1, week 1, month 1, 3, 6, 9, 12, 18. BCVA and C/D were inspected every three months postoperatively. The visual field test was repeated at 12 months after the surgery and MD was compared with baseline data. Up to now, all patients were followed up for at least 12 months. Goldmann applanation tonometer (BQ 900, Haag-Streit AG, Sweden) was used to measure IOP. Optometry unit (Refraction Table HRT-7000, Huvitz, Korea) and LogMAR units were applied to test spherical equivalent (SE) and BCVA. Fundus photograph (Digital Retinal Camera CR-2 AF, Canon, Japan) was taken to record C/D. The visual field was tested with 30–2 SITA-Fast strategy (Humphrey® Field Analyzer, Model 750i, Carl Zeiss Meditec Inc. Dublin, CA. USA). Slit-lamp (BQ 900, Haag-Streit AG, Sweden) was adopted to observe complications.

Efficacy was assessed by the success rate of circumferential catheterization, IOP values, the success rate of the modified surgery, and the number of IOP-lowering agents. Circumferential catheterization with a twisted 6/0 suture was deemed successful cannulation. The success of the modified surgery was defined on three criteria: postoperative IOP ≤ 21 mmHg, 18 mmHg, 15 mmHg, and ≥ 20% IOP reduction. Complete success includes patients who reached the criteria without medical therapy, while qualified success includes those meeting specific IOP reduction level with or without medical therapy. IOP-lowering medication were given when the IOP was over 18 mmHg. Safety was evaluated by intraoperative and postoperative adverse events. Moreover, supportive outcomes included BCVA, C/D, and MD.

### Statistical analysis

Normally distributed variables were presented as mean ± standard deviation. Analysis of Variance (ANOVA) was used to compare the differences between preoperative and postoperative data collected. Other variables were presented as medium with range, and non-parametric tests (Wilcoxon test) were used. Kaplan–Meier analysis was performed to calculate cumulative rates of success. Cox regression analysis was used to explore factors influencing the success rates of the modified technique. Value for *p* < 0.05 was considered statistically significant. SPSS Statistics 23.0 (Statistical Product and Service Solutions, IBM, USA) was applied for analyzing statistics.

## Results

### Characterization of cannulation materials

The pictures of three candidates for SC probing are presented in Fig. 1, and corresponding parameters were listed in Table 1. After cauterization, the tip of the 5/0 suture was mushroom-shaped with a rough and sharp margin. The tip size was bigger than other two candidates, and its size varied depending on cauterization energy. The twisted 6/0 suture and iTrack™ had a very smooth tip and similar body diameter. The loop tip diameter of the twisted 6/0 suture was similar to SC and bigger than the blunt tip of iTrack™. The contact angle represents the affinity of the material for water. The contact angle of polypropylene material was 150°(Fig. 1f) which demonstrated that polypropylene sutures are hydrophobic, thus it could maintain stability in the SC during the cannulation. FTIR results showed that the polypropylene suture and iTrack™ are polymers with carbon chains (Fig. 1g). Meanwhile, the FTIR spectrum of iTrack™ indicates the presence of some hydrophilic groups.

**Table 1:** Comparison of physical parameters of probing materials

Parameters	5/0 suture	Twisted 6/0 suture	iTrack™
Maximum diameter of the tip (μm)	390	290	250
Maximum diameter of the body (μm)	150	200	200
Water contact angle (°)	150	150	N/A

*N/A* not applicable

### Baseline parameters

A total of 40 eyes of 40 consecutive patients were enrolled and received modified suture canaloplasty. Circumferential catheterization of SC by a twisted 6/0 polypropylene suture was successfully performed in 36 eyes of 36 patients (90%). Demographic and baseline parameters are summarized in Table 2.

**Table 2:** Demographic and baseline data

Characteristics	Total	Successful catheterization	Failed catheterization
No. of patients	40	36	4
No. of eyes	40	36(90%)	4(10%)
Gender			
Male/Female	36/4	33/3	3/1
Side			
Right/Left	20/20	20/16	0/4
Age at surgery (year)			
Mean ± SD	47.4 ± 9.9(34–70)	47.6 ± 10.3(34–70)	45.3 ± 6.0(40–53)
Follow-up time (month)			
Mean ± SD	15.0 ± 3.0 (12–18)	14.8 ± 3.0 (12–18)	16.5 ± 3.0 (12–18)
C/D			
Mean ± SD	0.86 ± 0.13 (0.6–1.0)	0.86 ± 0.13 (0.6–1.0)	0.88 ± 0.19 (0.6–1.0)
BCVA (LogMAR)			
Mean ± SD	0.62 ± 0.74 (-0.08 ~ 2.6)	0.61 ± 0.72 (-0.08 ~ 2.6)	0.74 ± 1.07 (-0.08–2.3)
SE (D)			
Mean ± SD	-4.35 ± -5.00 (0 ~ -18)	-4.56 ± -5.17 (0 ~ -18)	-2.50 ± -2.89 (0 ~ -5)
MD (dB)			
Mean ± SD	-15.75 ± 7.40 (-29.13 ~ -1.21)	-16.50 ± 6.99 (-29.13 ~ -2.42)	-7.27 ± 7.94 (-16.25 ~ -1.21)
Preoperative IOP (mmHg)			
Mean ± SD	26.8 ± 7.6 (22–50)	26.2 ± 6.9 (22–48)	32.0 ± 12.8 (22–50)
No. of preoperative medications			
Mean ± SD	3.2 ± 0.6(2–4)	3.2 ± 0.6(2–4)	3.0 ± 0.0(3–3)

*NO* number, *C/D* cup-to-disc ratio, *BCVA* best-corrected visual acuity, *LogMAR* log of the minimum angle of resolution, *SE* spherical equivalent, *IOP* intraocular pressure

### IOP, success rate, and the number of IOP-lowering agents

**Fig. 4:**
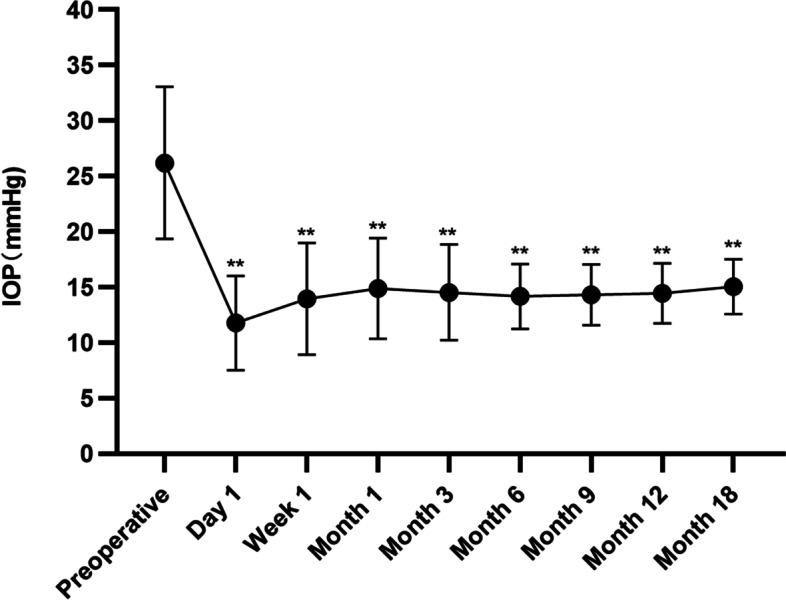
Intraocular pressure (IOP) before and after modified suture-assisted canaloplasty (**p < 0.001)

Preoperative and postoperative IOP and anti-glaucoma agents are exhibited in Table 3. The IOP decreased significantly after surgery (*F* = 33.12, *p* < 0.001). The preoperative and postoperative IOP curve is presented in Fig. 4.

**Table 3:** Preoperative and postoperative IOP and anti-glaucoma agents

Time	IOP (mmHg) Mean ± SD (range)	IOP reduction	Number of agents Mean ± SD (range)	n
Preoperative	26.2 ± 6.9 (22–48)		3.2 ± 0.6 (2–4)	36
1 day	11.8 ± 4.3 (6–21)	55.0%	0	36
1 week	13.9 ± 5.0 (7–35)	46.9%	0	36
1 month	14.9 ± 4.5 (8–35)	43.1%	0.6 ± 0.9 (0–3)	36
3 months	14.5 ± 4.3 (8–35)	44.7%	0.6 ± 0.9 (0–3)	36
6 months	14.2 ± 2.9 (9–21)	45.8%	0.5 ± 0.8 (0–2)	35
9 months	14.3 ± 2.7 (9–20)	45.4%	0.5 ± 0.8 (0–2)	35
12 months	14.5 ± 2.7 (9–20)	44.7%	0.5 ± 0.8 (0–2)	35
18 months	15.1 ± 2.5 (10–18)	42.4%	0.8 ± 0.9 (0–2)	17
*p*-value	< 0.001*		< 0.001*	

*IOP* intraocular pressure, *SD* standard deviation, 1 mmHg = 0.133 kPa, *n* sample size

^*^Repeated measured ANOVA

**Fig. 5:**
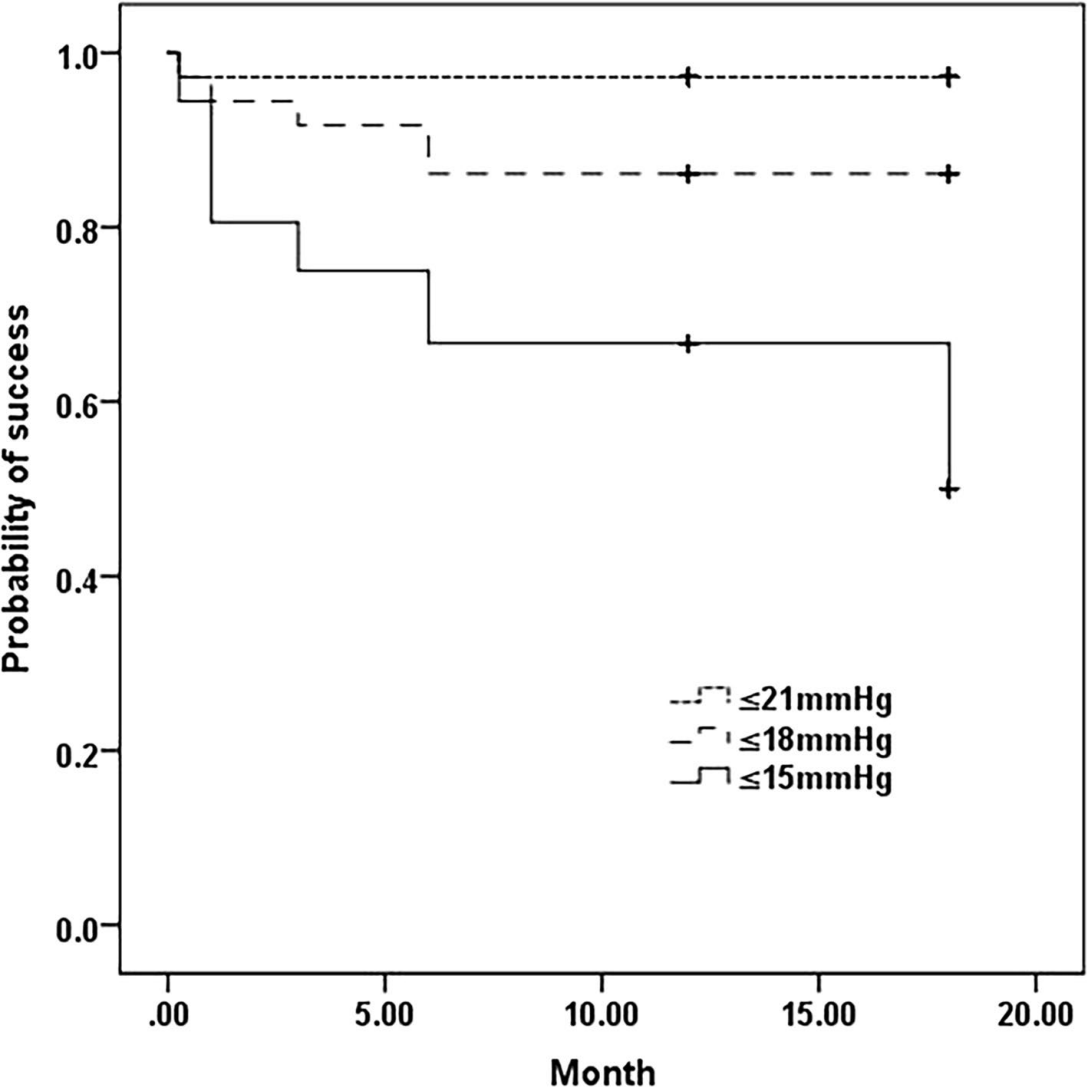
Kaplan–Meier survival plot of cumulative probability of qualified success with intraocular pressure (IOP) ≤ 21, 18 and 15 mmHg and ≥ 20% IOP reduction

At 12 months, qualified success rates were 97.2% [95% confidence interval (CI): 0.92–1.03], 86.1% (CI: 0.74–0.98) and 66.7% (CI: 0.51–0.83) with an IOP ≤ 21 mmHg, 18 mmHg, 15 mmHg and ≥ 20% reduction, respectively. Complete success rates were 66.7% (CI: 0.51–0.83), 61.1% (CI: 0.44–0.78) and 52.8% (CI: 0.36–0.70). The Kaplan–Meier survival analysis is displayed in Fig. 5. Age (β = 0.01, *p* = 0.04), preoperative IOP (β = 0.03, *p* = 0.003) and SE (β = 0.00, *p* = 0.01) negatively influenced the success rate significantly when the success was defined as an IOP ≤ 18 mmHg. Gender, side of the eyes, baseline BCVA, MD and the number of anti-glaucoma agents did not correlate significantly with any success rate.

The mean number of IOP-lowering agents decreased significantly from 3.2 ± 0.6 (2–4) preoperatively to 0.5 ± 0.8 (0–2) at 12 months postoperatively (*p* = 0.01). One patient received Ahmed valve implantation at 3 months after modified suture-assisted canaloplasty because of medically uncontrolled IOP. IOP was 17 mmHg at 12 months after Ahmed valve implantation.

### BCVA, C/D, and MD

Ten (25%) of the included patients had high myopia with SE ≤ -5D. SE was -4.35 ± -5.00D (0 ~ -18D). BCVA was not affected significantly by the surgery. One (2.8%) patient had a transient and mild postoperative visual acuity reduction of 1 to 2 lines. BCVA returned to the preoperative level at 1-month follow-up. There was no significant difference between preoperative C/D and postoperative C/D at each visit. For visual field parameters, MD at 12 months did not get worse and were not statistically significant (*p* = 0.323).

### Complications

As far as complications were concerned, hyphema occurred in 11 (30.6%) eyes during or after the operation and cleared within 1 week. Three patients (8.3%) had an IOP spike (IOP > 25 mmHg) at the early stage after surgery. In those patients,IOP returned to normal levels within 1 month. One patient (2.7%) had peripheral anterior synechia to the TDM. No other complications such as Descemet’s membrane detachment, choroidal detachment, or hypotony were observed (Table 4).

**Table 4:** Complications during and after the surgery

Complications	n(%)
Hyphema	11(30.6)
IOP spike	3(8.3)
Peripheral anterior synechia to the TDM	1(2.7)

*IOP* intraocular pressure, *TDM* trabecular-Descemet’s membrane

In 4 eyes of 4 patients who failed for circumferential cannulation because of intra-canal resistance or getting lost and entering the wrong way, only viscocanalostomy was performed. The IOP reduced from 32.0 ± 12.8 mmHg to 16.8 ± 7.3 mmHg and the number of IOP-lowering medication was reduced from 3.0 ± 0 to 0.5 ± 1.0 at 12 months after modified canaloplasty.

## Discussions

This prospective study provided clinical evidence to support modified suture-assisted canaloplasty as an effective and safe alternative to canaloplasty using a flexible microcatheter in Asian POAG patients. This is the first study evaluating the efficacy and safety of modified suture-assisted canaloplasty for the treatment of Asian patients with POAG. Moreover, we demonstrated that the twisted 6/0 suture possessed similar physical and chemical properties to the microcatheter apart from the illuminated tip.

Besides the cost factor, the twisted 6/0 polypropylene suture was an ideal material for SC cannulation in terms of characterization. With the help of material specialists, three candidates for probing were compared. The twisted 6/0 suture was selected as a prober based on the material evidence. The microcatheter costs 170 times more than the polypropylene suture. The sharp margin in the cauterized tip of the 5/0 suture affected the circumferential cannulation. However, twisted 6/0 sutures possessed a smooth loop tip and similar body diameter to iTrack™. It also had a deformable loop tip helping to advance in the SC, which was better than iTrack™ and avoided false pathing[10]. Material characterizations indicate that the polypropylene suture has similar physical and chemical properties to iTrack™, although some differences exist such as without illuminated tip.

Three modifications were made to improve the efficacy of canaloplasty, including SC opening by viscocanalostomy, circumferential probing by a twisted 6/0 suture, and loose suture of the superior scleral flap. Attributed to our experience of viscocanalostomy for over twenty years [7, 14, 16], SC was accurately located during the dissection of the deep scleral flap. Twisted 6/0 suture was selected for its superior physical and chemical properties, which were demonstrated in the previous part. The superficial scleral flap was sutured in a loose way for external outflow of the aqueous humor. Canaloplasty is a new surgery aiming to restore the natural outflow way of aqueous humor by two mechanisms[17]. One is to extend SC and distal outflow by viscoelastic substance. The other is to persistently dilate the SC by placing a double-strand 10–0 polypropylene suture in the canal. Circumferential viscodilation of SC alone is also called canaloplasty or ab-interno canaloplasty[18, 19]. However, persistent suture tension was not included, which could be the key to sustained IOP reduction.

As is well known, the key of the modified procedure was successful circumferential cannulation of SC. In this study, the success rate of circumferential cannulation by twisted 6/0 sutures was 90%. Most of the trials studying canaloplasty were assisted by iTrack™. However, the success rate of 360°intubation varied from one to another. Previous studies by Lewis showed success rates of 360°cannulation were 78.7% [17] and 74% [20]. Hughes reported a probing rate of 80.9% by Visco360 or Omni System [21], Bull reported a successful probing of 89.9% [22], and Brusini reported a probing rate of 90.8% [23]. Furthermore, Xin reported a success rate of 89.2% [24]. Afterward, a few doctors applied sutures in canaloplasty instead of a dedicated microcatheter. Haus initiated the twisted 6/0 suture for circumferential probing and reported a probing rate of 71.6% [10]. Our previous study reported a cannulation rate of 88.7% by 5/0 suture in primary congenital glaucoma. In this study, the probing rate was higher than most of the studies of canaloplasty aided by either the illuminated microcatheter or suture. One possible reason is the surgeon’s experience of viscocanalostomy for over twenty years [14]. Viscocanalostomy helped to open SC accurately when making the deep scleral flap, which guaranteed the success of 360°cannulation. The other reasons are the smooth loop tip and flexibility of the twisted 6/0 polypropylene suture. In our study, circumferential cannulation failed in 4 patients (10%). Failed cannulation owed to adhesion, occlusion, and incorrect passage of probing material in SC [25].

Significant IOP reduction was achieved at each follow-up visit postoperatively. For an IOP ≤ 21, ≤ 18, and ≤ 15 mm Hg and ≥ 20% reduction, our result presented qualified success rates of 97.2%, 86.1%, and 66.7%, and complete success rates of 66.7%, 61.1%, and 52.8% at 12 months respectively. Viscocanalostomy distended only a third to half of SC and distal outflow way of aqueous humor, while canaloplasty could circumferentially expand SC. Thus, canaloplasty could achieve 2 mmHg of IOP reduction compared with viscocanalostomy[4]. Seuthe et al. reported complete success rates of 65.8% and 59.5% with an IOP ≤ 21 mmHg and 18 mmHg [26]. Vastardis reported that complete and qualified success rates were 74.31% and 90% with an IOP ≤ 21 mmHg [27]. Furthermore, Vastardis reported an absolute success rate of 20.19% in advanced POAG [28]. The reason for the low success rate could be the definition of the success rate (5 ≤ IOP ≤ 15 mmHg), which was lower than usual criteria (IOP ≤ 21 mmHg or IOP ≤ 18 mmHg). For IOP ≤ 21 mmHg, ≤ 18 mmHg and ≤ 16 mmHg, Brusini achieved a qualified success rate of 92.1%, 84.3%, and 68.5%, while a complete success rate of 70.8%, 67.4%, and 59.5% at 2 years postoperatively [23]. Our results presented superior IOP-lowering efficacy to most studies. This achievement may be due to combining canaloplasty and viscocanalostomy which supplied more drainage ways through TDM. Besides internal outflow through distended SC, the aqueous humor was drained out in several ways after TDM. Some went to the SC through both of the ostia, some was absorbed by the new aqueous humor veins on the sclera, some went through the uveoscleral outflow, and the remaining traveled by the subconjunctival path through loosely sutured scleral flap [1, 4].

Cox regression analysis showed that age, preoperative IOP, and SE negatively influenced the success rate significantly in this study. Grieshaber et al. reported younger age positively influenced IOP-lowering efficacy while preoperative IOP did not [29]. Thus, early surgical intervention was suggested because of a better prognosis. Hughes reported that high preoperative IOP positively influences the amount of IOP reduction [21], which was contrary to our study. Enrolled patients in Hughes’s study were diagnosed with mild to moderate glaucoma, while most of the patients were advanced glaucoma in our study. We supposed that the natural outflow pathway was more damaged and less likely to be restored in advanced POAG. Also, higher myopia predicted the failure of canaloplasty because myopia is associated significantly with POAG [30].

The number of IOP-lowering medications decreased from 3.2 ± 0.6 preoperatively to 0.5 ± 0.8 postoperatively significantly. The number of medications was similar to or less than previous studies [11, 31]. For high IOP after modified canaloplasty, laser goniopunture was adopted to make one or two tiny holes to increase queous humor from the anterior chamber to the scleral lake under the superficial scleral flap. Kodomskoi et al. applied laser goniopuncture (LGP) in 18% of the patients, and iris incarceration occurred in 4% [11]. LGP was performed in 9.9% of the patients in a study by Brusini et al. In this study, LGP was not adopted to avoid breaking the 10/0 polypropylene suture kept in the SC for dilating the canal.

In terms of complication, hyphaema was the most common and was cleared within 1 week postoperatively. Hyphaema was retrograde bleeding from episcleral venous because of dilated SC. It was deemed a predictor of surgical success because it indicated permeability of the distended trabecular meshwork and distal outflow of aqueous humor [32]. Another complication was transient high IOP. It stemmed from viscoelastic material [25]. In our study, the occurrence of IOP spike was 8.3% which is lower than other studies because viscocanalostomy supplied external outflow of aqueous humor at the early stage after surgery[10, 26, 32–34]. Only one patient had mild peripheral anterior synechia to the TDM. Neither Descemet’s membrane detachment nor cyclodialysis occurred in our study [35].

Potential limitations need to be considered in our study. Firstly, blind cannulation aided with the twisted 6/0 polypropylene suture could lead to complications due to a wrong turn. Secondly, the follow-up period was limited. Given the novel nature of this modified surgery, 12-month data was the best we could achievable. The long term follow-up is still on going. Thirdly, the sample size was small with a single-center study which may cause statistical bias. A multi-center study with a large sample size should be planned for rigorous analysis. Ideally, a head-to-head controlled study between suture-assisted canaloplasty and microcatheter-assisted canaloplasty will be needed to demonstrate their efficacy and safety in circumferential catheterization and the management of glaucoma.

In summary, we presented that the twisted 6/0 suture can be an ideal material for SC cannulation. Modified suture-assisted canaloplasty could achieve effective IOP reduction and decreased medication burden with few complications and low cost. Modified suture-assisted canaloplasty seems to be a promising, cost-efficient, and accessible alternative to microcatheter-assisted canaloplasty.

## Supplementary Information

**Additional file 1. **Original data.

## Data Availability

The datasets used and/or analyzed during the current study are available from. the corresponding author on reasonable request.

## Abbreviations

POAG: Primary open-angle glaucoma
SC: Schlemm’s canal
IOP: Intraocular pressure
PCG: Primary congenital glaucoma
FTIR: Fourier transform infrared
BCVA: Best-corrected visual acuity
C/D: Cup-to-disc ratio; MD: Median deviation
SE: Spherical equivalent
TDM: Trabecular-Descemet’s membrane
ANOVA: Analysis of Variance
CI: Confidence interval
LGP: Laser goniopuncture
